# Efficacy and safety of transarterial chemoperfusion for head and neck malignancies

**DOI:** 10.1007/s11547-025-02039-2

**Published:** 2025-07-04

**Authors:** Thomas J. Vogl, Andrea Tröger, Simon Bernatz, Timo Stöver, Hamzah Adwan

**Affiliations:** 1 Clinic for Radiology and Nuclear Medicine, University Hospital Frankfurt, Johann Wolfgang Goethe University, Theodor-Stern-Kai 7, 60590 Frankfurt, Germany; 2 Department of Otorhinolaryngology, University Hospital Innsbruck, Medical University Innsbruck, Anichstrasse 35, 6020 Innsbruck, Austria; 3 Department of Otorhinolaryngology, University Hospital Frankfurt, Johann Wolfgang Goethe University, Theodor-Stern-Kai 7, 60590 Frankfurt, Germany

**Keywords:** Head and neck cancer, Interventional radiology, Transarterial chemoperfusion, TACP

## Abstract

**Purpose:**

To evaluate tumor response and overall survival (OS) of patients with head and neck malignancies treated by transarterial chemoperfusion (TACP).

**Materials and methods:**

This monocentric retrospective study included a total of 77 patients with head and neck malignancies, who were at least treated by one session of TACP in palliative-intent between 2002 and 2021. Tumor response to therapy was assessed using the Response Evaluation Criteria in Solid Tumors (RECIST). Local control was defined as achieving either partial response (PR) or stable disease (SD).

**Results:**

All 77 enrolled patients (mean age: 59 ± 12 years; 52 males) were analyzed according to OS. RECIST was performed in 70 out of 77 patients since follow-up imaging was not available in 7 patients. No major complications were observed. Considering RECIST, PR was achieved in 48.57% and SD in 44.29% of the patients, resulting in a local control rate of 92.86%. A total of 5 patients showed progressive diseases at a rate of 7.14%. The median OS was 12.6 ± 1.5 months. Patients with T1-T3 tumors had significantly longer OS compared to patients with T4.

**Conclusion:**

Palliative TACP has the potential to improve local tumor control and achieve remarkable survival outcomes for patients with head and neck area malignancies.

## Introduction

Head and neck cancer ranks globally as the sixth most common cancer, accounting for approximately 630,000 new cancer cases annually, and it is the seventh leading cause of death worldwide [1, 2]. It is a heterogeneous group of tumors of the oral cavity, pharynx, larynx, nose, and salivary glands. While potentially curative therapies, such as surgery, radiation therapy, and chemotherapy, have demonstrated success, palliative therapy can be necessary depending on the tumor state at baseline diagnosis or during follow-up. These patients may suffer from severe symptoms such as pain, dysphagia, dyspnea, and dysgeusia [3].

Palliative systemic chemotherapy is used to reduce these symptoms and aims to extend the patient’s survival. Although multiple regimens have been tested, systemic application of treatment can lead to multiple side effects. Additionally, dose-dependent toxicity can determine the upper limit of dosage, which is a potential reason why systemic chemotherapy has limits of local doses of chemotherapy within the tumor area [3, 4]. However, chemotherapy can also be applied locally. It differs from intravenous systemic chemotherapy by allowing higher doses of chemotherapeutic agents to be delivered directly to the tumor. This more localized drug delivery approach minimizes exposure of healthy tissues to cytotoxic agents, thereby reducing the risk of systemic adverse effects, when compared to systemic chemotherapy [5–7].

Transarterial chemoperfusion (TACP) is a method for the regional application of chemotherapy. TACP capitalizes on the unique vascular anatomy of head and neck tumors, which often receive their blood supply from branches of the external carotid artery. By selectively catheterizing these arterial branches under angiographic control, interventional radiologists can deliver high doses of chemotherapy directly into the tumor bed. Although TACP is more recognized as a possible treatment modality for liver and lung cancer [8–12], its application in the head and neck region remains infrequent. To date, TACP in the head and neck area has demonstrated notable effectiveness, especially in a palliative context, without serious side effects [8, 13, 14]

However, TACP is not used regularly in patients with head and neck malignancies. Therefore, this study aims to investigate tumor response and patients’ survival following palliative TACP in the head and neck region.

## Materials and methods

### Study population

This retrospective study, approved by the Institutional Review Board (No. 2023–1111), identified patients with palliative head and neck cancer who underwent at least one TACP procedure between 01/2002 and 03/2021. Data collected included patient demographics, tumor location, histology, TNM stages, complications, and chemotherapeutic agents. Patients receiving palliative TACP for unresectable/recurrent head and neck tumors and tumors unresponsive to prior therapies were included. TACP aimed to control the tumor, extend life, and alleviate symptoms such as dyspnea, dysphagia, and dysphonia.

Informed consent was obtained before each session.

Exclusion criteria included lack of initial digital staging CT or MRI, concurrent therapies in other organs, thrombosis in the common carotid artery or external carotid artery, anemia, leukopenia, and coagulopathy.

### TACP procedure

All therapies were administered on an outpatient basis and repeated at 4 week intervals until tumor progression, patient withdrawal, exclusion criteria were met, or the patient’s general condition deteriorated.

Chemotherapeutic agents were selected based on individual assessments by a multidisciplinary tumor board, considering prior exposure to cytostatic agents, tumor type, age, and concurrent medical conditions. Dosages of all chemotherapeutics followed manufacturer recommendations and were individualized based on body surface area, with the chemotherapy solution volume adjusted accordingly (between 30 and 50 ml).

The chemotherapy agents used included: mitomycin C (8 mg/m^2^; mito-medac®, Medac), irinotecan (100 mg/m^2^; Irinotecan Aurobindo®, PUREN Pharma GmbH), gemcitabin (800 mg/m^2^; Gemcitabin HEXAL®, Hexal AG), cisplatin (35 mg/m^2^; Cisplatin Accord, Accord Healthcare Limited), docetaxel (40 mg/m^2^; docetaxel-ratiopharm®, Ratiopharm GmbH) and paclitaxel (100 mg/m^2^; Abraxane®, Bristol Myers Squibb). Table 1 shows the different combinations of chemotherapeutic agents used.

**Table 1:** Combinations of chemotherapeutic agents

Chemotherapeutic agents	n	Percentage %
Mitomycin C	2	2.6
Mitomycin C + Gemcitabine	6	7.8
Mitomycin C + Cisplatin	3	3.9
Cisplatin + Docetaxel	1	1.3
Cisplatin + Gemcitabine	1	1.3
Mitomycin C + Gemcitabine + Cisplatin	49	63.6
Mitomycin C + Irinotecan + Cisplatin	14	18.2
Mitomycin C + Cisplatin + Paclitaxel	1	1.3

TACP was performed as follows: After local anesthesia, catheterization was conducted through the femoral or brachial artery using the Seldinger technique. An initial angiography, using a pigtail catheter and contrast agent, was used to visualize the arteries supplying blood to the tumor. Next, selective catheterization followed using a cobra, sidewinder, or headhunter catheter, placing the catheter in at least one main tumor-feeding branch of the external carotid artery. If multiple arteries supplied the tumor, the catheter was repositioned as needed. Once the catheter reached the target arteries, another angiography series was performed using a contrast agent to confirm proper placement. During this step, the interventional radiologist looked for a “blush”—a visible contrast enhancement within the tumor on the angiography images, indicating blood flow into the tumor. This confirmed that the catheter was correctly positioned for delivering chemotherapy. If no blush appeared, meaning the contrast agent was not reaching the tumor, the catheter was repositioned until the proper placement was achieved. The chemotherapy solution was then manually injected by the interventional radiologist using controlled pressure. If high resistance was encountered, the process was briefly paused and then resumed. This method ensured that a high concentration of the drug reached the tumor while remaining tolerable for the patients. The procedure typically took about one hour and was used for both primary tumor masses and large lymph node masses.

### Follow-up imaging

Before each TACP, MRI scans of the head and neck, including native and contrast-enhanced T1 and T2 images, were performed. Post-interventional CT scans of the head and neck ruled out complications. This imaging sequence was repeated at every TACP, occurring every 4 weeks.

An experienced radiologist (*.*., > 20 years) evaluated all imaging. Therapy response was assessed using the Response Evaluation Criteria in Solid Tumors (RECIST) [15] and categorized as complete response (CR), partial response (PR), stable disease (SD), or progressive disease (PD). While RECIST identifies lymph nodes with a short axis of ≥ 15 mm as malignant, we categorized lymph nodes with a short axis of ≥ 10 mm as malignant, given the established understanding that lymph nodes with a short axis of ≥ 10 mm are typically malignant in the head and neck region [15, 16].

Therapy response was measured by changes in tumor volume before therapy and at each TACP session. Volume changes between sessions were attributed to the preceding TACP. Manual volumetric segmentation was performed using a slice-by-slice approach with the AW Workstation software (GE Healthcare, Vienna, Austria). The tumor borders were delineated using the ‘paint on slice’ tool, and the software then calculated the volumes of the segmented tumors upon completion.

### Complications

Complications were assessed through post-TACP head and neck CT imaging and patient records. They were classified using the “Society of Interventional Radiology’s classification system for complications by outcome” into minor and major complications. Minor complications required no or minimal treatment without lasting consequences, while major complications required treatment, hospitalization, and caused permanent damage, or led to death.

### Statistical analysis

Statistical analysis was performed using ‘Statistical Package for the Social Sciences’ version 26.0.0.0 (SPSS Statistics; IBM), with a significance level of *α* = 0.05. Descriptive statistics included means and medians. The distribution of the data was assessed using the Shapiro–Wilk test for normality. Based on the test results, normally distributed data are presented as mean ± standard deviation (SD), while non-normally distributed data are reported as medians.

Survival was analyzed with the Kaplan–Meier estimator from the first TACP to death or last contact.

Predictive factors were assessed using the log-rank test for categorical variables and Cox regression for continuous variables, including patient sex, tumor histology, TNM classification, tumor location, recurrence status, number of TACPs, therapeutic response, and chemotherapeutic agents. Combinations of multiple factors were tested with linear regression, and differences between variables were evaluated using single-factor analysis of variance (ANOVA).

## Results

### Patients

**Fig. 1:**
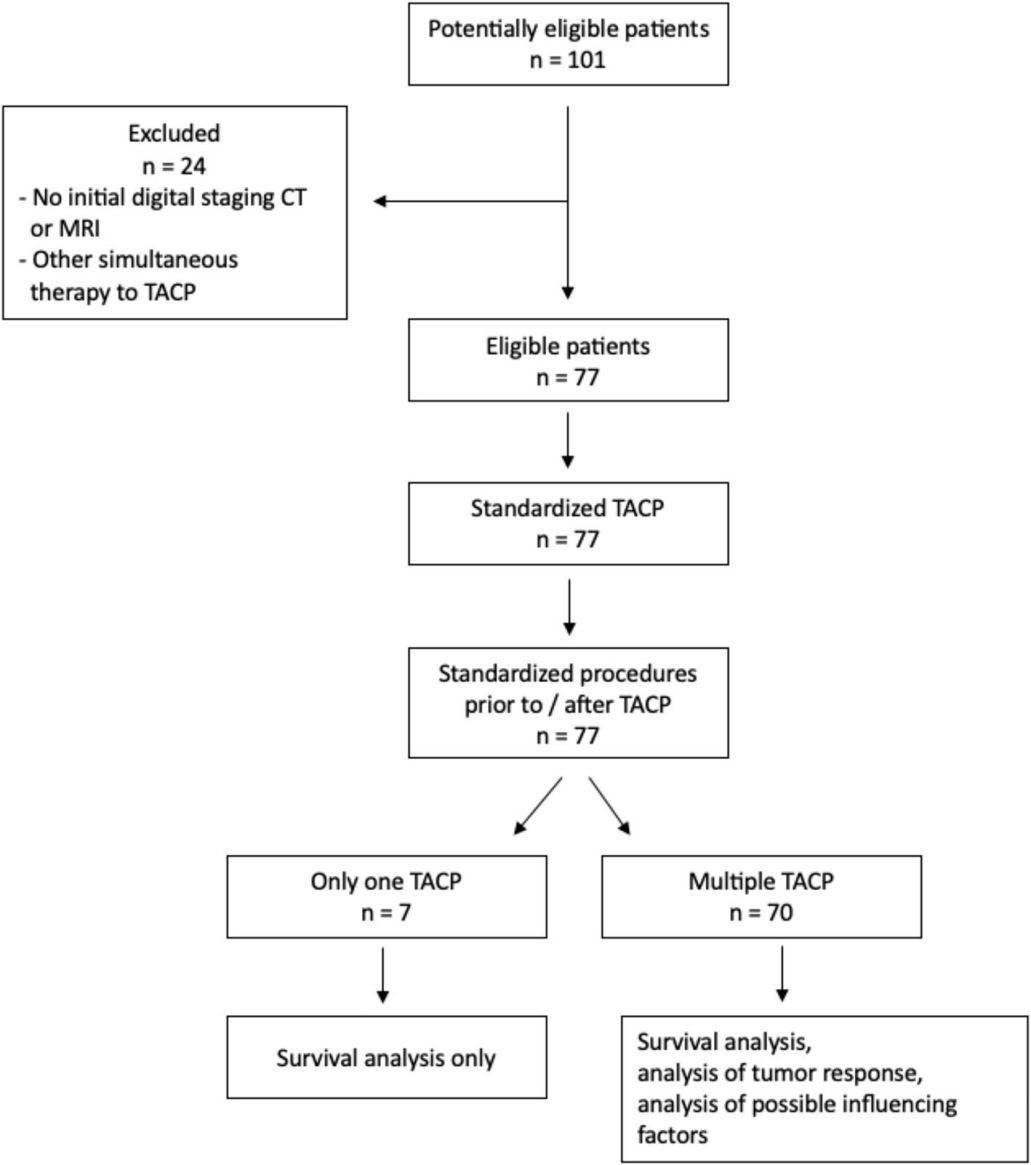
A flow chart of the study process is shown

After applying the inclusion and exclusion criteria, as seen in Fig. 1, this study included 77 patients (mean age 59 ± 11.9 years; 52 males and 25 females). Their characteristics are shown in Table 2. Patients received between 1 and 13 TACPs, with a median of 4 (interquartile range (IQR) 2) TACPs. In total, 300 TACPs were evaluated.

**Table 2:** Patient characteristics

Variables	n	Percentage %
*Gender*		
Male	52	67.5
Female	25	32.5
*Mean age*		
Male	58.9 years	
Female	60.2 years	
*Tumor histology*		
Squamous cell carcinoma	69	89.6
Others (adenocarcinoma, mucoepidermoid carcinoma)	8	10.4
*Primary tumor site*		
Nasopharynx	2	2.6
Oropharynx	28	36.4
Oral cavity	9	11.7
Hypopharynx	10	13.0
Larynx	6	7.8
Salivary glands	7	9.1
Nasal cavity and sinuses	2	2,6
Cervical lymph node recurrence	13	16.9
*T-state of TNM-Classification*		
T0	12	15.6
T1	2	2.6
T2	10	13.0
T3	17	41.2
T4	36	46.8
*N-state of TNM-Classification*		
N0	28	36.4
N1	13	16.9
N2	29	37.7
N3	7	9.1
*M-state of TNM-Classification*		
M0	58	75.3
M1	19	24.7
*Number of TACPs per patient*		
1 TACP	7	9
2 TACPs	17	22.1
3 TACPs	14	18.2
4 TACPs	15	19.5
≥ 5 TACPs	24	31.2

#### Adverse events

No major complications were observed. Among the minor complications, a total of 25 patients (32.5%) experienced nausea during the intervention, which was treated with an intravenous injection of granisetron. The rate of patients who had fatigue 1–2 days after TACP was 40.3% (31/77). No cases of vomiting were observed.

#### Tumor response

Response to treatment was evaluated in 70 of 77 patients since the follow-up imaging was not available in seven patients.

**Fig. 2:**
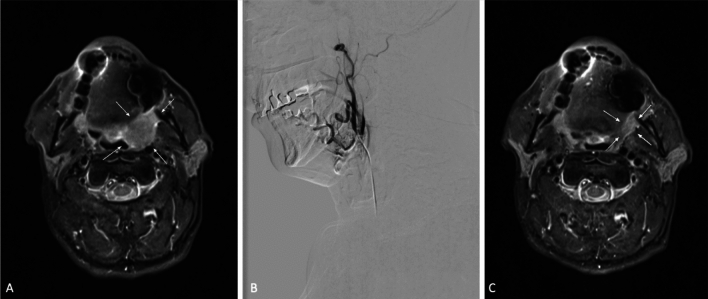
The case of a 61 year-old male with oropharyngeal squamous cell carcinoma is presented. Image **A** shows the MRI scan of the tumor prior to therapy. Image B displays the selective catheterization of the tumor-feeding branches of the external carotid artery. A combination of mitomycin c, gemcitabin, and cisplatin was administered. Image **C** shows the MRI scan of the tumor after multiple TACP sessions. The arrows point to the tumor. Overall, a PR was achieved, with a 60.46% reduction in tumor volume

The change in tumor volume was assessed. Based upon serial measurements and observations, in 65 of the 70 patients, 93% of the tumors regressed to some extent during the treatment. Regression of greater than 30% was noted in 50% of the patients and regression of less than 30% was observed in 43% of the patients. At the time of the best treatment response, the mean tumor volume reduction was 34.86 ± 28.1%. Figure 2 shows an example of a patient's response to therapy. Following the first TACP session, there was a mean volume reduction of 19.39 ± 2.7%. Overall, each TACP session resulted in a mean volume reduction of 16.16 ± 17.53%.

A statistically significant positive correlation was found between the reduction of tumor volume following the first TACP session and the tumor volume reduction observed at the time of the best response to therapy (adjusted R-squared = 0.441, regression coefficient = 0.852, *p* < 0.001).

Similarly, patients with a high mean tumor volume reduction per TACP session showed a greater reduction in tumor volume at the time of best treatment response. This correlation was statistically validated (corrected R-squared = 0.421, regressions coefficient = 1.050, *p* < 0.01).

No statistically significant effects on the overall response were observed based on gender, patient age, histology of the tumor, TNM classification, tumor location, or initial tumor volume.

**Fig. 3:**
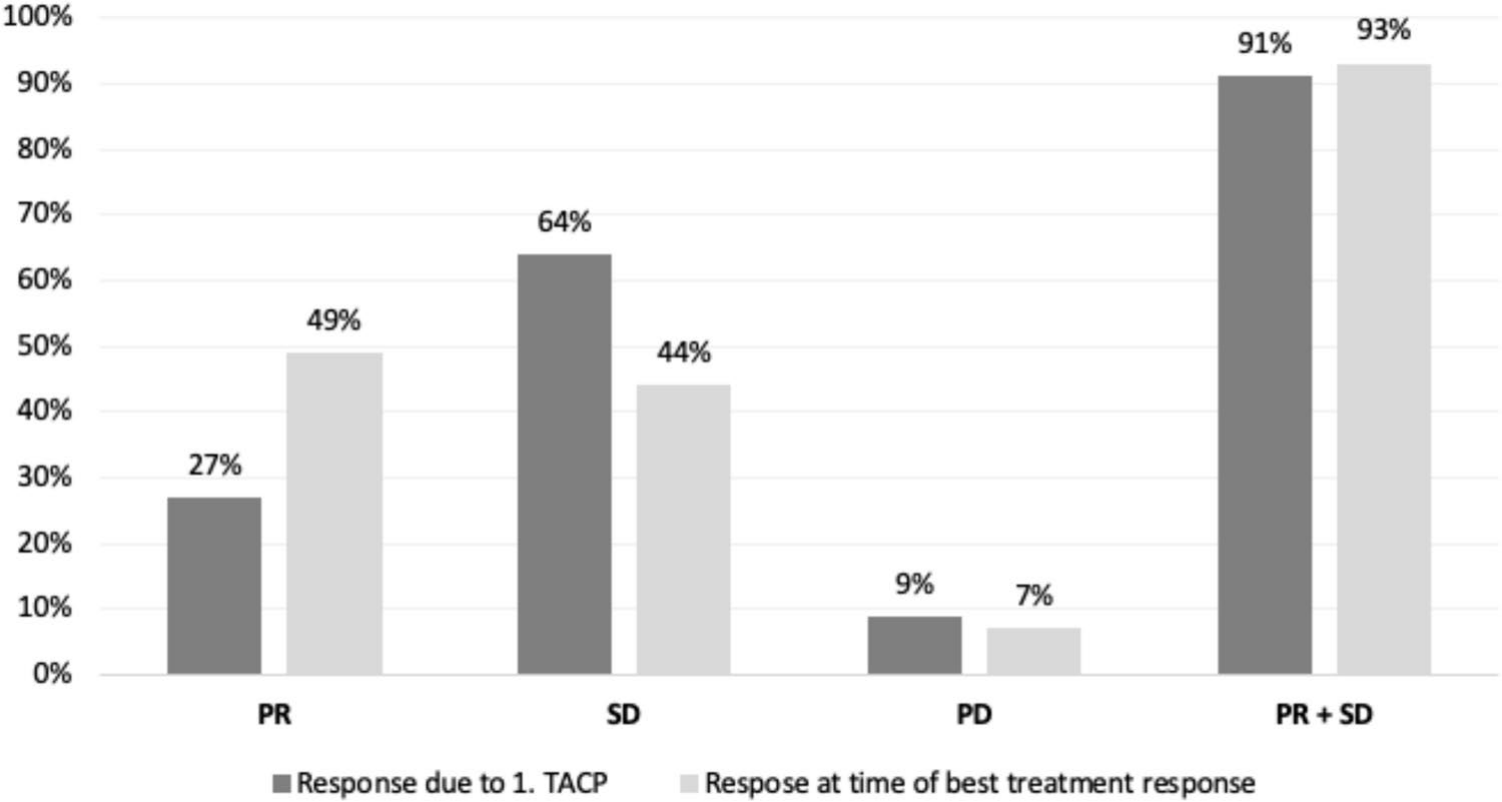
The figure shows tumor responses to therapy after the first TACP and at the time of the best response in each case. Following the first TACP, a favorable response was observed, with 27% of cases achieving a PR and 64% exhibiting SD. As treatment progressed, the response distribution shifted, with many tumors that initially had SD subsequently achieving a PR

Considering the RECIST, PR was reached in 49% (*n* = 34) of the patients, 44% (*n* = 31) of the patients achieved SD, and 7% (*n* = 5) of the patients showed PD at time of best therapy response. As a result, local control of the tumor was achieved in 93% (*n* = 65) of the cases. Figure 3 depicts the tumor responses graphically.

#### Survival

A total of 39 (50.6%) patients died, while the remaining 38 patients (49.4%) were censored at the last contact. In the overall survival analysis, the median overall survival (OS) for all patients was 12.6 ± 1.5 months (IQR 14.9; 95% CI 9.6–15.6 months). The OS rates at 6, 12, and 24 months were 68.1%, 52.9%, and 31.6%, respectively.

**Fig. 4:**
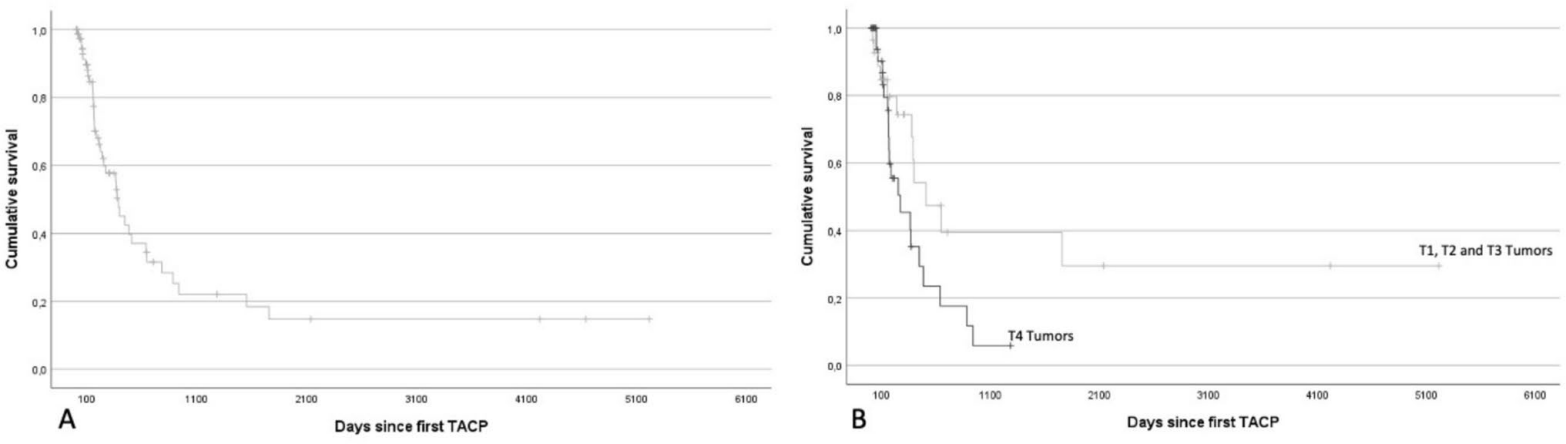
Graph A shows the cumulative survival of all patients, and Graph **B** shows the cumulative survival sorted by T stage of the tumor

**Fig. 5:**
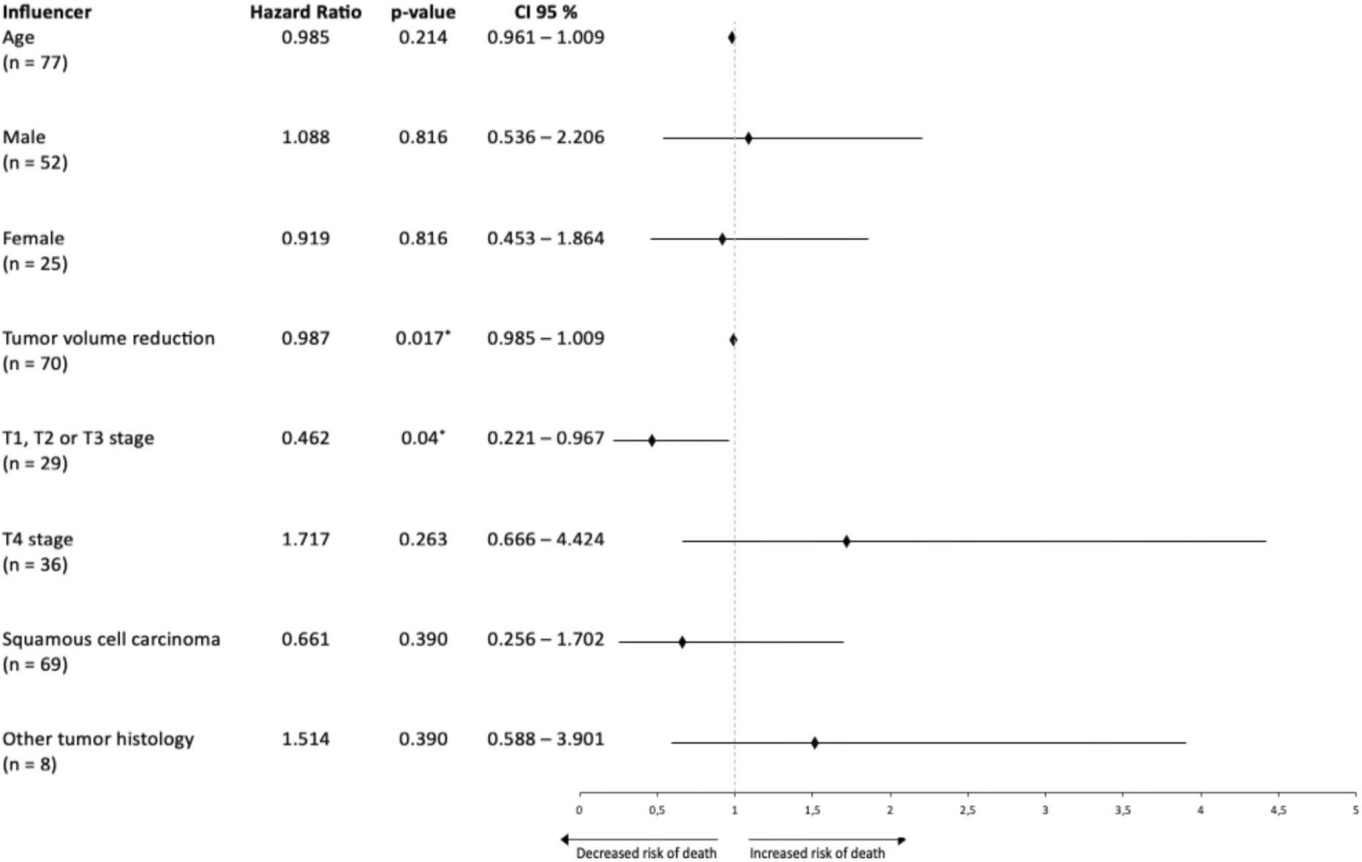
A forest plot depicting the results of a Cox proportional hazards model. The plot displays the HR along with their corresponding CI 95% for each covariate examined in the model. The diamonds on the plot represent the HR, while the lines extending from the diamonds represent the CI 95% for each covariant. The asterisk after the *p*-values indicates significance

Using the log-rank (Mantel-Cox) test, a statistically significant effect of the T stage of the tumor on OS was found. Patients with tumors in the T1, T2, or T3 stage at the onset of therapy displayed significantly longer survival time compared to those patients with tumors in the T4 stage (*p*-value = 0.036). Patients with a lower T stage had a median OS of 16.5 ± 4.86 months (IQR 9; 95% CI 7–26.1 months), while those patients with T4 stage tumors showed a median survival of 8.8 ± 3.95 months (IQR 3.9; 95% CI 1.1–16.6 months). Figure 4 shows the corresponding Kaplan–Meier survival chart. The influence of the T stage was additionally tested with a Cox proportional hazard analysis, where a lower T stage reduced the risk of dying and, therefore, led to longer survival (hazard ratio 0.462 Gender, age, and tumor histology did not show a significant influence on survival in the Cox regression analysis. Figure 5 summarizes the results of the Cox regression.

## Discussion

TACP is a recognized procedure with various applications [9–12], though its use in the head and neck region remains relatively uncommon, despite existing studies in the literature [13, 14, 17].

In this study, while focusing on palliative TACP for head and neck cancer, promising results were observed with 48.57% PR and 44.29% SD. These results compare favorably to other studies, such as Donegan et al., who reported tumor regression in 87% of the patients [17], and Huntington et al., who reported a 62% response rate (CR, PR, and SD) [14]. Given that in our study, 93% of the tumors regressed and local control was defined as PR and SD, the response rate here appears slightly favorable. However, unlike other studies [14, 17], we did not observe any CR, which may be attributed to the heterogeneity in patient populations with varied prior therapies and tumor pathologies, making it challenging to create comparable patient groups.

The variability in survival times may also reflect the mixed patient group. Rohde et al. reported a 1-year survival rate of 21% after TACP [13], while our study achieved 52.9% with a median OS of 12.6 months. Differences in cisplatin dosage may account for this, with Rohde et al. using 150 mg/m^2^ compared to our 35 mg/m^2^. Additionally, in Rohde’s study, all patients received TACP with cisplatin, whereas in our study, this was the case for 90% of patients [13]. Higher doses of chemotherapeutic agents can help overcome drug resistance of tumors. Cisplatin doses vary across studies, up to 200 mg/m^2^ of body surface area [18–20]. Robbins et al. showed that 150 mg/m^2^ per week is well tolerated with a cisplatin neutralizer like sodium thiosulfate [21]. As no neutralizer was used in the present study, a lower dose was necessary. Unlike intravenous administration, where drug delivery depends on cardiac output to the tumor-feeding artery, local arterial application ensures that nearly all drug molecules reach the tumor. The smaller the tumor-feeding artery, the lower the percentage of cardiac output it receives, resulting in fewer drug molecules reaching it. Therefore, selective transarterial drug administration seems to be advantageous for tumors supplied by small blood vessels. Local factors such as tissue blood flow, capillary permeability, binding, and partition coefficients do not significantly influence the effectiveness of intra-arterial versus intravenous drug administration [5].

Bahl et al. reported a median OS of 8 months with platinum-based palliative chemotherapy for head and neck tumors [22]. In contrast, this study achieved a slightly higher median survival of 12.6 months. While cisplatin-based chemotherapy is common, recent evidence suggests that monoclonal antibodies like cetuximab or pembrolizumab may extend survival [22–24]. Several studies have reported a median survival range of 10.1–13 months [22–25]. Monoclonal antibodies were not used in the present study, but they may become part of future studies.

Locally applied chemotherapy, primarily resulting in tumor size reduction, has been shown to improve function, alleviate pain, and increase survival [26–28]. In this study, the average tumor mass reduction was 34.86%, potentially improving swallowing, speech, and breathing. While no significant association in risk (HR of 0.987) between tumor volume reduction and survival time was found, the therapeutic goal of improving patient well-being remains crucial. Thus, side effects should be minimized during palliative therapy [26, 29–32].

Immediate and major complications following TACP were evaluated via CT scan, revealing no major complications. However, no standardized follow-up was conducted to assess functional improvements in swallowing, breathing, and speech. A multicenter study by Ackerstaff et al. found that patients receiving TACP experienced less nausea, vomiting, and fatigue compared to intravenous chemotherapy [26]. The direct administration of chemotherapy into the tumor-feeding artery during TACP may lead to a reduced systemic impact, which could be the reason for this effect. However, the presumably lower systemic effect of TACP may not always be beneficial. There is debate about whether TACP reaches peripheral organs. If it does not, intravenous chemotherapy might be superior for addressing potential micrometastases and would thus suppress further development of new metastases [8, 33–36]. TACP may be preferable for patients who are sensitive to systemic side effects or those patients with poor overall health. Additionally, patients with inadequate responses to intravenous chemotherapy might benefit from TACP, as localized delivery allows higher doses, potentially overcoming tumor resistance to chemotherapy [8, 37].

It is important to understand that this study has some limitations, particularly due to its retrospective design and missing data, especially on prior treatments, metastasis timing, immunohistochemical analysis of the tumor, and post-therapy treatments. A major criticism is the lack of follow-up and unexplained therapy discontinuations, preventing the calculation of time to progression. Nevertheless, despite these limitations, the findings provide additional data to the limited body of literature on palliative TACP for head and neck cancer. The results indicate that TACP may contribute to local tumor control and have the potential to support survival in selected patients. Further research is needed to validate these findings and better define the role of TACP in future treatment strategies.

In conclusion, TACP is a viable approach for managing palliative head and neck cancer, offering potential benefits in local tumor control and survival extension. Our findings support wider implementation in the head and neck area, though further research is needed to address study limitations.

## Abbreviations

CI 95%: 95% Confidence interval
CR: Complete response
HR: Hazard ratio
IQR: Interquartile range
OS: Overall survival
PD: Progressive disease
PR: Partial response
RECIST: Response Evaluation Criteria in Solid Tumors
SD: Stable disease
STD: Standard deviation
TACP: Transarterial chemoperfusion
